# Preferential Use of Central Metabolism *In Vivo* Reveals a Nutritional Basis for Polymicrobial Infection

**DOI:** 10.1371/journal.ppat.1004601

**Published:** 2015-01-08

**Authors:** Christopher J. Alteri, Stephanie D. Himpsl, Harry L. T. Mobley

**Affiliations:** Department of Microbiology and Immunology, University of Michigan Medical School, Ann Arbor, Michigan, United States of America; Osaka University, Japan

## Abstract

The human genitourinary tract is a common anatomical niche for polymicrobial infection and a leading site for the development of bacteremia and sepsis. Most uncomplicated, community-acquired urinary tract infections (UTI) are caused by *Escherichia coli*, while another bacterium, *Proteus mirabilis*, is more often associated with complicated UTI. Here, we report that uropathogenic *E. coli* and *P. mirabilis* have divergent requirements for specific central pathways *in vivo* despite colonizing and occupying the same host environment. Using mutants of specific central metabolism enzymes, we determined glycolysis mutants lacking *pgi*, *tpiA*, *pfkA*, or *pykA* all have fitness defects *in vivo* for *P. mirabilis* but do not affect colonization of *E. coli* during UTI. Similarly, the oxidative pentose phosphate pathway is required only for *P. mirabilis in vivo*. In contrast, gluconeogenesis is required only for *E. coli* fitness *in vivo*. The remarkable difference in central pathway utilization between *E. coli* and *P. mirabilis* during experimental UTI was also observed for TCA cycle mutants in *sdhB, fumC*, and *frdA*. The distinct *in vivo* requirements between these pathogens suggest *E. coli* and *P. mirabilis* are not direct competitors within host urinary tract nutritional niche. In support of this, we found that co-infection with *E. coli* and *P. mirabilis* wild-type strains enhanced bacterial colonization and persistence of both pathogens during UTI. Our results reveal that complementary utilization of central carbon metabolism facilitates polymicrobial disease and suggests microbial activity *in vivo* alters the host urinary tract nutritional niche.

## Author Summary

The human urinary tract is a leading source for polymicrobial infections and for the development of bacteremia and sepsis. Treating these potentially dangerous infections have recently become more challenging due to the appearance of uropathogenic strains that are resistant to the many of the most commonly prescribed antibiotics. The majority of urinary tract infections (UTI) are caused by *Escherichia coli*, while another bacterium, *Proteus mirabilis*, is more likely to cause catheter-associated UTI. Here, we report that uropathogenic *E. coli* and *P. mirabilis* have divergent nutritional requirements despite growing in the same host environment. This result indicates that *E. coli* and *P. mirabilis* do not directly compete for nutrients during UTI. Indeed, we found that persistence of both pathogens is enhanced when they co-colonize the host. This work represents an important step toward understanding the basic nutritional requirements for two major pathogens that cause UTI and shows how mixed infections can change these requirements. Understanding how bacteria grow during infections is fundamental to ultimately uncover new ways to combat increasingly drug-resistant bacterial infections.

## Introduction

The recent revival of interest in the relationship between bacterial metabolism and host-pathogen interactions has deepened our understanding of pathogen colonization and growth *in vivo* [1], [2], [3], [4]. Consequently, central metabolism must be considered essential to virulence because bacterial pathogens must use nutrients available within the host niche to cause disease [5], [6]. The relationship between the pathogen's available carbon and energy sources, or host nutritional niche [7], and pathways required for replication *in vivo* has been demonstrated for a variety of pathogenic microbes. Extraintestinal pathogenic *E. coli* require peptide import systems, the TCA cycle, and gluconeogenesis to consume amino acids and peptides in the urinary tract [8], while intestinal pathogenic *E. coli* require pathways to catabolize multiple sugars available in the intestine [9]. *Salmonella enterica* serovar Typhimurium exploits the inflammatory response of the host that creates an alternative electron acceptor to allow the pathogen to respire and compete with anaerobic gut residents [10]. The energetic consequences of modulating respiratory chain components and proton motive force can also promote pathogen survival in the face of bactericidal activities of the host [11].

The contribution of central carbon pathways to pathogenesis has been shown for numerous intracellular and extracellular nutritional niches occupied by pathogens. Expression-based and genetic approaches using the model cytosolic pathogen, *Listeria monocytogenes*, indicate that gluconeogenesis and uptake and catabolism of glycerol and dihydoxyacetone are required for bacterial replication *in vivo* [12], [13], [14], [15]. These findings are supported by the observation that disruption of glucose uptake has no effect on *L. monocytogenes* intracellular replication [16], suggesting that glycerol is the preferred carbon source for *Listeria in vivo*. *Shigella flexneri*, which also replicates in the host cell cytosol, requires glycerol-3-phosphate as a carbon source [17]. Interestingly, enteroinvasive *E. coli* (EIEC), which are genetically-related to *S. flexneri*, also use 3-carbon substrates as a carbon source *in vivo*. Because chorismate, GMP, and thymidylate synthesis have been found essential for *S. flexneri* replication *in vivo* [18], [19] and *de novo* synthesis of amino acids is extensive, it is believed that glycerol metabolism and anabolic pathways in this bacterium and EIEC may be important for bacteria that replicate within epithelial cells during intestinal infection.

*Mycobacterium tuberculosis* that replicates intracellularly within phagocytes, utilizes fatty acids in addition to glycerol or glycerol-3-phosphate as a carbon source in macrophages [20]. Seminal studies show that *in vivo* carbon metabolism in *M. tuberculosis* is dependent on fatty acid catabolism and the glyoxylate shunt through the TCA cycle [21], [22], [23]. The collective requirement for glycerol catabolism for *L. monocytogenes*, *S. flexneri*, EIEC, and *M. tuberculosis* growth *in vivo* likely indicates that glycerol is a readily available carbon source inside host cells and extends Rolf Freter's nutrient-niche hypothesis [7] to include available nutrients within a eukaryotic cell. However, despite occupying a similar host microenvironment, intracellular glycerol is not the preferred carbon source for *S. enterica* serovar Typhimurium because glucose import, glycolysis, and the oxidative TCA cycle are required for *Salmonella* to colonize the intestine and replicate within host phagocytes [24], [25], [26], [27]. This key difference in preferred carbon source *in vivo* could reflect a *Salmonella* fitness adaptation for facing increased competition with a diversity of luminal gut anaerobes.

Extracellular or luminal colonizers, including both commensal and pathogenic *E. coli*, are able to occupy the host gastrointestinal tract, yet their nutritional requirements for carbon metabolism *in vivo* have key differences. Colonization studies using the streptomycin-treated mouse model in combination with transcriptional profiling during culture in mucus demonstrated that the ED pathway, and gluconate or other sugar acids, are required for intestinal growth of commensal *E. coli* [28]. EHEC requires similar central metabolic pathways as commensal strains, however, EHEC colonization *in vivo* requires the catabolism of up to six additional sugars [9]. EHEC also utilizes glycolytic substrates and switches to gluconeogenic substrates when present in the intestine with commensal *E. coli*, which solely utilizes glycolytic pathways for *in vivo* growth [29]. This finding, that competition *in vivo* can alter preferred routes of carbon flux through the central pathways, introduces the notion that studying polymicrobial interactions during host colonization is essential to understand the relationship between bacterial metabolism and pathogenesis.

For extraintestinal pathogenic *E. coli* (ExPEC), it has been shown that D-serine metabolism and acetogenic growth are important during colonization of the urinary tract [30], [31]. In previous work, we have demonstrated that the import of peptides, gluconeogenesis, and the TCA cycle are required for *E. coli* during extraintestinal infection, while glycolysis and the pentose phosphate pathway are dispensable [8]. This indicates *E. coli* has to synthesize sugars from amino acids (gluconeogenesis) while enzymes for sugar catabolism have no affect on fitness. Although less is known about the *in vivo* metabolism of *Proteus mirabilis*, another important urinary tract pathogen, it would be expected to have the same enzymatic requirements during infection. Attenuated strains of *P. mirabilis* have been identified with mutations in genes that encode proteins involved in gluconate and pyruvate metabolism, and in enzymes of the TCA cycle, using signature-tagged mutagenesis [32], [33]. These earlier studies are supported by a recent comparison of global gene expression studies from *E. coli* [34], [35] and *P. mirabilis* [36] that indicated many similarities and some subtle differences may exist *in vivo* between these uropathogens during experimental infection of the urinary tract. To better understand the relationship between the host nutritional niche and pathogen growth, we used defined mutants, each defective in specific metabolic pathways, to directly examine the *in vivo* metabolism for two bacterial pathogens that occupy the same host niche and likely have access to the same nutrients during infection.

**Figure 1:**
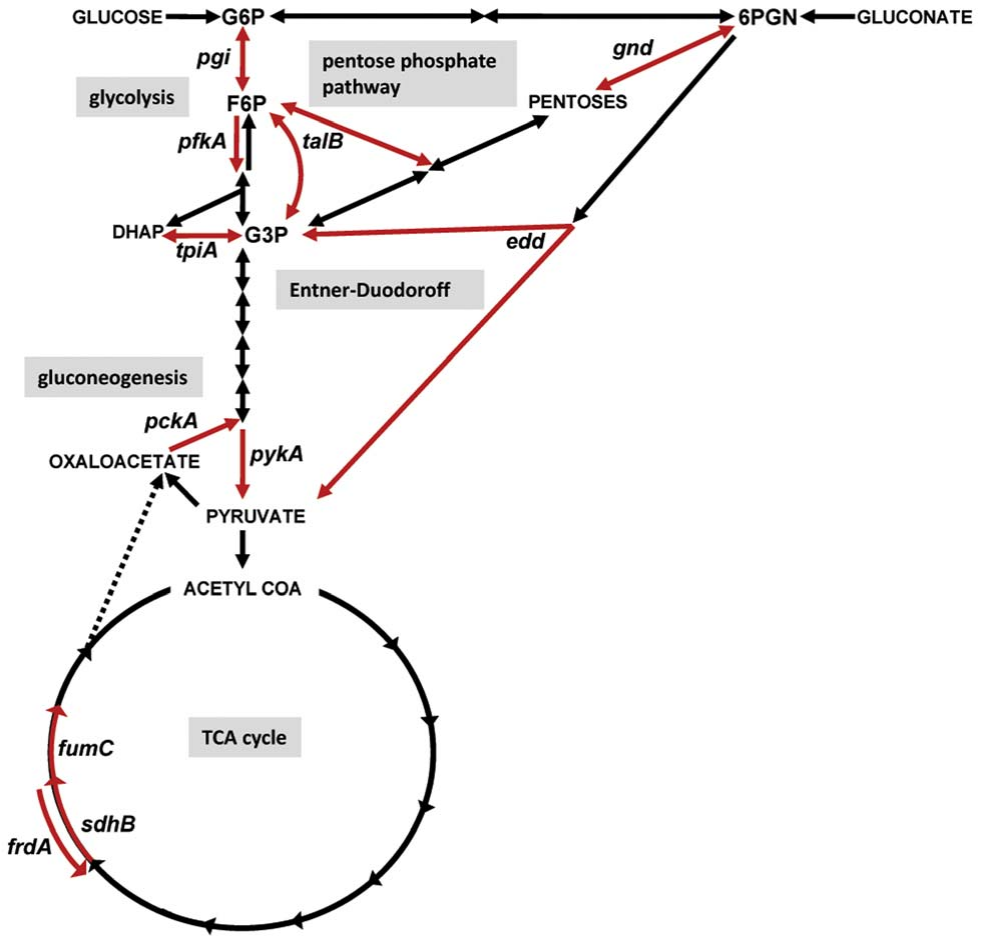
Diagram of central metabolism and map of the specific pathways disrupted by targeted mutations in uropathogenic *E. coli* and *P. mirabilis*. Carbon sources or biochemical intermediates shared between pathways are indicated in capital letters or abbreviated: G6P, glucose-6-phosphate; F6P, fructose-6-phosphate; G3P, glyceraldehyde-3-phosphate; 6PGN, 6-phosphogluconate. Reactions are denoted with arrows. Specific reactions (red arrows) were targeted by deletion or insertion in *E. coli* CFT073 or *P. mirabilis* HI4320, respectively. In glycolysis: *pgi*, glucose-6-phosphate isomerase; *pfkA*, 6-phosphofructokinase transferase; *tpiA*, triosephosphate isomerase; *pykA*, pyruvate kinase; in pentose phosphate pathway: gnd, 6-phosphogluconate dehydrogenase; *talB*, transaldolase; in Entner-Duodoroff pathway: *edd*, 6-phosphogluconate dehydratase; in gluconeogenesis: *pckA*, phosphoenolpyruvate carboxykinase; and in the TCA cycle: *sdhB*, succinate dehydrogenase; *fumC*, fumarate hydratase; *frdA*, fumarate reductase.

Unexpectedly, we found remarkably divergent *in vivo* requirements for central pathways between these two pathogens during UTI by assessing the *in vivo* fitness of strains containing mutations in *pgi*, *pfkA*, *tpiA*, *pykA*, *gnd*, *talB*, *edd*, *sdhB*, *fumC*, *frdA*, and *pckA*, in both uropathogenic *E. coli* CFT073 and *P. mirabilis* HI4320 (Fig. 1). Because the urinary tract is a normally sterile environment, in this current study, we not only further characterized the role of central metabolism during host colonization for both pathogenic *E. coli* and *P. mirabilis* in mono-species infection, but it was also possible to develop a polymicrobial infection model using the host urinary tract as an unoccupied vessel. Using this new model and, as their complementary utilization of central pathways suggested, we found that co-infection with *E. coli* and *P. mirabilis* wild-type strains enhanced bacterial colonization and persistence of both pathogens during UTI. These findings help explain the molecular and biochemical basis of polymicrobial infection in the urinary tract.

## Results

### Glycolysis is required for *P. mirabilis* and dispensable for *E. coli* during urinary tract infection

**Figure 2:**
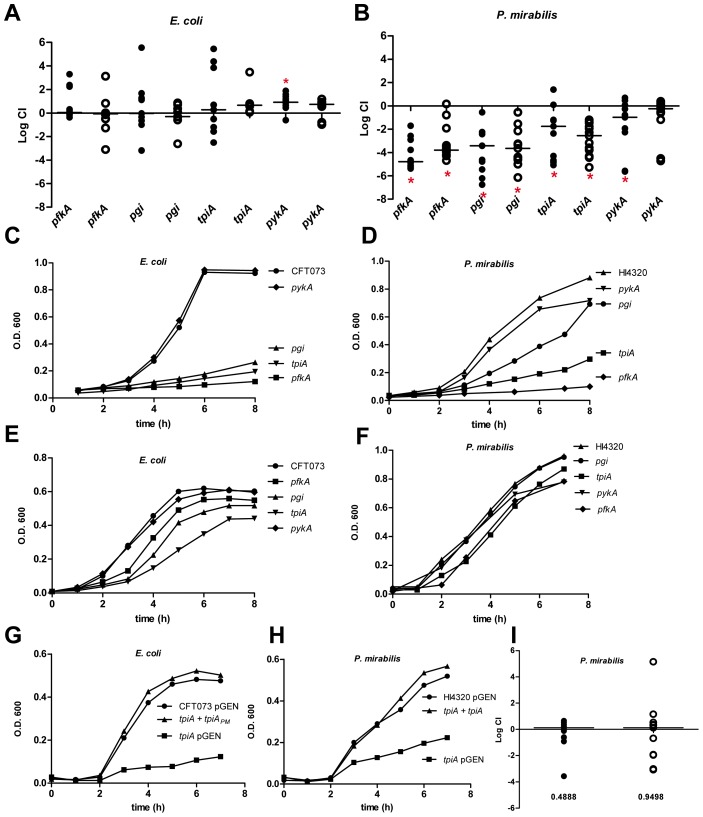
Glycolysis is required for *P. mirabilis* but is dispensible for *E. coli* during experimental urinary tract infection. *In vivo* competitive indices (CI) were determined following co-challenge infections of susceptible female CBA/J mice with a 1∶1 ratio of either wild-type (A) *E. coli* CFT073 or (B) *P. mirabilis* HI4320 and their respective glycolysis mutant strains. *E. coli* were recovered at 48 h post-inoculation (hpi). *P. mirabilis* was recovered at 7 d post-inoculation (dpi). Each dot represents bladder (closed symbols) and kidneys (open symbols) from an individual animal. Bars indicate the median CI. Significant differences in colonization (*P<0.05) were determined with the Wilcoxon signed-rank test. A CI<1 indicates a fitness defect. Growth of (C, E) *E. coli* CFT073 or (D, F) *P. mirabilis* HI4320 wild-type strains and mutants in: *pgi*, glucose-6-phosphate isomerase; *pfkA*, 6-phosphofructokinase transferase; *tpiA*, triosephosphate isomerase; and *pykA*, pyruvate kinase during culture in defined medium containing 0.2% glucose as the sole carbon source (C, D) or in LB medium (E, F). A representative growth curve is shown for each panel. Restoration of wild-type growth in triosephosphate isomerase mutants (G) growth in MOPS defined medium containing 0.2% glucose of *E. coli* CFT073 (pGEN), UPEC *tpiA* (pGEN), and the *E. coli* CFT073 *tpiA* mutant containing pGEN into which the *tpiA* gene from *P. mirabilis* was cloned. (H) *P. mirabilis* HI4320 containing empty vector (pGEN), HI4320 *tpiA* (pGEN), and complemented HI4320 *tpiA* mutant containing pGEN-*tpiA* in minimal salts medium containing 0.2% glucose. (I) *In vivo* complementation of *P. mirabilis tpiA* mutant bacteria. Competitive indices were determined following an *in vivo* co-challenge infection of female CBA/J mice with *P. mirabilis* HI4320 (pGEN) and HI4320 (*tpiA* pGEN-*tpiA*). Bacteria were recovered at 7 dpi. Each dot represents the CI in bladders (closed symbols) and kidneys (open symbols) from an individual animal. Bars represent the median CI. P-values are indicated on the graph.

Despite extensive biochemical and *in vitro* studies of the model organism *E. coli*, characterization of central carbon pathways for extraintestinal pathogenic *E. coli* during infection is considerably less well understood than well-considered virulence factors [6]. Recently, a uropathogenic isolate was used to investigate pathogenic *E. coli* central metabolism in an infection model and found that in contrast to commensal *E. coli*, glycolysis is dispensable for extraintestinal pathogenic *E. coli* during colonization, while gluconeogenesis is required during infection [8]. One limitation from that study is the glycolytic enzymes investigated in that work could also play a role in gluconeogenesis. To address this, additional glycolytic mutants were constructed in *E. coli* CFT073, a prototype strain isolated from the blood and urine of a patient with acute pyelonephritis and urosepsis [37], [38]. In addition to strains lacking *tpiA* (triose phosphate isomerase) and *pgi* (phosphoglucose isomerase), mutants in irreversible glycolytic steps involving both 6-carbon (*pfkA*; 6-phosphofructokinase transferase) and 3-carbon (*pykA*; pyruvate kinase) substrates were constructed and tested in competitive infections with the parental *E. coli* CFT073 strain. Using the well-established murine model of ascending infection [39], we found that disruption of either the preparative or substrate level phosphorylation stages of glycolysis had no effect on the ability of *E. coli* to compete with wild-type CFT073 during experimental infection (Fig. 2A).

Because the growth medium within the urinary tract, urine, is a dilute mixture of amino acids and peptides [40], it is not unexpected that glycolysis would be dispensible during UTI. The composition of urine and its relative lack of available carbohydrates (except under diabetic condition), along with the lack of a significant contribution of glycolysis for *E. coli* during experimental UTI, predicts that glycolysis mutants in *P. mirabilis*, another common urinary tract pathogen, would have no apparent fitness defect *in vivo*. To test this, mutations were constructed in *P. mirabilis* HI4320, in the same glycolysis genes tested for *E. coli*: *pgi*, *pfkA*, *tpiA*, and *pykA*. Unexpectedly, any mutation that disrupted glycolysis in *P. mirabilis* resulted in a significant fitness defect using the same model of ascending infection (Fig. 2B). With the exception of *pykA* that demonstrated a fitness defect in the bladder (*P* = 0.031), all of the remaining *P. mirabilis* glycolysis mutants tested were out-competed by wild-type parental HI4320>1,000-fold (*P*<0.050) in both bladders and kidneys (Fig. 2B).

The dramatic differential requirement for glycolysis during infection between *E. coli* and *P. mirabilis* was not due to differences during *in vitro* growth. In both *E. coli* and *P. mirabilis*, only mutants in *pfkA*, *pgi*, and *tpiA* demonstrated the expected growth defect in defined medium containing glucose as the sole carbon source; growth rates of *pykA* mutants in defined medium containing glucose was similar to wild-type for both strains (Fig. 2C and D). Both sets of glycolysis mutants also demonstrated growth rates similar to the parental strains in LB medium (Fig. 2E and F). It was possible to complement the *in vitro* growth defect for the *P. mirabilis tpiA* mutant by introducing the *tpiA* gene from wild-type HI4320 into the mutant strain on a low copy plasmid (Fig. 2H). The re-introduction of the wild-type *tpiA* allele also restored the ability of the mutant strain to colonize the urinary tract; *in vivo* complementation resulted in a complete reversal of the *tpiA* fitness defect during competitive infection with the parental wild-type HI4320 harboring an empty vector control plasmid (Fig. 2I). It was also possible to heterologously complement the growth defect for the *E. coli tpiA* mutant in defined medium containing glucose by introduction of the wild-type *tpiA* allele cloned from *P. mirabilis* HI4320 (Fig. 2G).

### *E. coli* and *P. mirabilis* transaldolase mutants have impaired fitness during UTI

**Figure 3:**
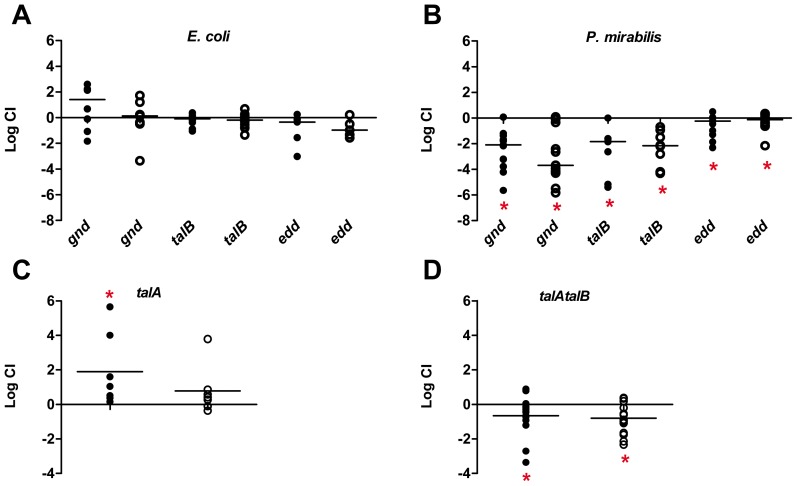
*In vivo* role for the pentose phosphate pathway and the Entner-Doudoroff pathway for pathogen colonization of the urinary tract. Competitive indices (CI) were determined following co-challenge infections of female CBA/J mice with a 1∶1 ratio of either wild-type (A) UPEC CFT073 or (B) *P. mirabilis* HI4320 and their respective mutants in the following genes: gnd, 6-phosphogluconate dehydrogenase; *talB*, transaldolase; and *edd*, 6-phosphoglyconate dehydrase. The contribution of multiple transaldolase isoenzymes in *E. coli* were assessed using co-challenge infections with wild-type *E. coli* CFT073 and (C) *talA* or (D) *talAtalB* mutant strains. *E. coli* CFT073 was cultured from bladders and kidneys at 48 hpi. *P. mirabilis* HI4320 was cultured from organs at 7 dpi. Each dot represents bladder (closed symbols) and kidneys (open symbols) from an individual animal. Bars indicate the median CI. Significant differences in colonization (*P<0.05) were determined by Wilcoxon signed-rank test. A CI<1 indicates a fitness defect.

Previously, it has been shown that mutation of *E. coli* transaldolase A gene (*talA*) does not negatively affect fitness during extraintestinal infection despite TalA being induced by CFT073 when cultured in human urine [8]. This suggests that the non-oxidative pentose phosphate pathway does not significantly contribute to pathogen fitness during urinary tract infection. To better characterize the contribution of the isomerizations of the non-oxidative pentose phosphate pathway *in vivo*, an additional transaldolase mutant, transaldolase B (*talB*), was constructed in *E. coli* CFT073. TalB is the major transaldolase in *E. coli* that transfers a three-carbon moiety from a C_7_ molecule to glyceraldehyde-3-P (C_3_) to form erythrose-4-P (C_4_) and fructose-P (C_6_). This stage of the pentose phosphate pathway is reversible, and thus, can be uncoupled from the oxidative decarboxylation reactions that produce NADPH. While loss of the major transaldolase, TalB, did not affect *E. coli* fitness during UTI (Fig. 3A); *P. mirabilis talB* mutant bacteria were out-competed>100-fold by wild-type in both the bladders and kidneys (*P*<0.003) (Fig. 3B).

One possibility for a difference between *E. coli* and *P. mirabilis* requirement in the non-oxidative pentose phosphate pathway is the redundancy of transaldolase in *E. coli* [41]. *P. mirabilis* strains encode a single transaldolase enzyme (TalB), while *E. coli* strains have both TalA and TalB, which catalyze identical reactions for the cell. To determine if the lack of a fitness defect for the *E. coli talB* mutant (Fig. 3A), is due to functional redundancy, we tested the *talA* single mutant and constructed and tested a *talA talB* double mutant strain in competitive infections with the parental CFT073 wild-type strain. In these studies, we found that lack of *talA* resulted in the wild-type being outcompeted by the single mutant in the bladder (*P* = 0.043) (Fig. 3C). Although not as striking as the *talB* mutant in *P. mirabilis*, loss of both *talA* and *talB* in *E. coli* resulted in the transaldolase double mutant being out-competed by wild-type CFT073>5.0-fold in bladders and kidneys (*P*<0.050) (Fig. 3D).

### Disruption of the Entner-Doudoroff pathway or NADPH production in the pentose phosphate pathway creates a fitness defect in *P. mirabilis* but does not affect *E. coli* fitness during UTI

To distinguish the relative importance of the oxidative branch of the pentose phosphate pathway from the non-oxidative transaldolase-containing branch, a mutant in phophogluconolactonate (*gnd*) and a mutant defective in gluconate catabolism, 6-phosphoglyconate dehydrase (*edd*), were tested in competitive infections with the parental CFT073 wild-type strain. *E. coli* lacking either the oxidative pentose phosphate pathway (*gnd*), or the ED pathway (*edd*) were recovered from bladders and kidneys in numbers not significantly different from the wild-type strain (median CI = 1) (Fig. 3A). Surprisingly, unlike the lack of contribution for oxidative production of NADPH in the pentose phosphate pathway for *E. coli, P. mirabilis* mutants in *gnd* were out-competed>100-fold by wild-type in both the bladders and kidneys (*P*<0.003) (Fig. 3B).

Previously, signature-tagged mutagenesis identified a *P. mirabilis edd* transposon insertion as attenuated during experimental UTI [33], however, the attenuation caused by the disruption of *edd* was not confirmed by testing a ‘clean’ isogenic mutant strain *in vivo*. Despite this, it was reasonable to expect that, similar to the different requirement for glycolysis between *E. coli* and *P. mirabilis* during infection, *P. mirabilis* may require the capacity to metabolize gluconate via the Entner-Duodoroff pathway *in vivo*. Consistent with this, we found that in contrast to the findings with *E. coli*, the *P. mirabilis edd* mutant was significantly out-competed in both the bladders and kidneys by the parental HI4320 strain (*P*<0.020) during co-challenge infections when co-inoculated 1∶1 with wild-type (Fig. 3B). With the exception of *talA*, both *E. coli* and *P. mirabilis* share the same complement of the Entner-Duodoroff and pentose phosphate genes; therefore it is unlikely that redundancy of transaldolase in *E. coli* can account for the disparate requirements for these pathways between the two pathogens. In support of this, the *edd*, *talA, talB*, *talAtalB*, and *gnd* strains in *E. coli* and the *edd*, *talB*, and *gnd* strains in *P. mirabilis* all demonstrate similar growth *in vitro* and also to both wild-type parental strains during culture in LB medium and defined medium containing glucose as the sole carbon source (S1 Fig.).

### *In vivo* contribution of the TCA cycle and gluconeogenesis during urinary tract infection

**Figure 4:**
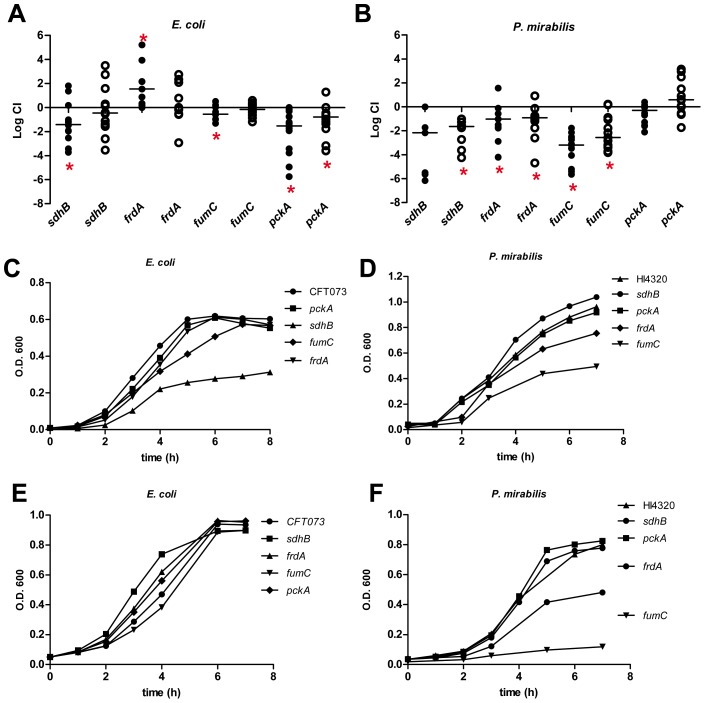
Contribution of the TCA cycle and gluconeogenesis during UTI. Competitive indices (CI) were determined following co-challenge infections of female CBA/J mice with a 1∶1 ratio of either wild-type (A) *E. coli* CFT073 or (B) *P. mirabilis* HI4320 and their respective mutants in the following genes: *sdhB*, succinate dehydrogenase; *fumC*, fumarate hydratase; *frdA*, fumarate reductase; and *pckA*, phosphoenolpyruvate carboxykinase. *E. coli* was cultured from bladders and kidneys at 48 hpi. *P. mirabilis* was cultured from organs at 7 dpi. Each dot represents bladder (closed symbols) and kidneys (open symbols) from an individual animal. Bars indicate the median CI. Significant differences in colonization (*P<0.05) were determined by the Wilcoxon signed-rank test. A CI<1 indicates a fitness defect. Growth of (C, E) *E. coli* CFT073 and (D, F) *P. mirabilis* HI4320 wild-type and mutant strains in: *sdhB*, *fumC*, *frdA*, and *pckA* in LB medium (C, D) or defined medium containing 0.2% glucose (E, F) as the carbon source. A representative growth curve is shown for each panel.

The aerobic tricarboxylic acid (TCA) cycle has been proposed to be required for *E. coli* fitness during growth on gluconeogenic substrates present in the urinary tract [8]. Specifically, *E. coli sdhB* mutant bacteria have been shown to have fitness defects during UTI [8], [42], suggesting that the reductive TCA cycle may not be operating during host colonization. To better define the role for the TCA cycle during extraintestinal infection, mutants of *E. coli* and *P. mirabilis* lacking succinate dehydrogenase; *sdhB*, fumarate dehydratase (fumarase); *fumC*, and fumarate reductase; *frdA* were constructed and tested in competitive infections with wild-type *E. coli* CFT073 or *P. mirabilis* HI4320, respectively. While both *E. coli* and *P. mirabilis* required TCA cycle reactions for fitness *in vivo*, *sdhB* was required for fitness only during cystitis (bladder CFU) in *E. coli* (Fig. 4A) and only during pyelonephritis (kidney CFU) in *P. mirabilis* (Fig. 4B) (*P*>0.050). It is generally believed that the production of reduced FADH_2_ during the conversion of succinate to fumarate by succinate dehydrogenase is avoided during fermentation by modification of the TCA cycle to an incomplete reductive pathway where fumarate conversion to succinate by fumarate reductase replaces succinate dehydrogenase activity. The loss of FrdA resulted in a fitness defect for *P. mirabilis* during infection of both the bladder and kidneys (*P*>0.005) (Fig. 4B). In contrast, *E. coli frdA* mutant colonization levels were indistinguishable from wild-type (median CI = 1.0) in the kidneys and significantly outcompeted the parental CFT073 strain>50-fold during acute cystitis (*P* = 0.024) (Fig. 4A). Both *E. coli* and *P. mirabilis* required *fumC*, which functions to convert fumarate to malate; loss of FumC, however, resulted in a severe fitness defect for *P. mirabilis* during bladder and kidney infection (CI<10^−3^, *P*<0.005), while the *E. coli fumC* mutant colonized the kidneys to similar levels as the parental CFT073 strain (CI = 0.94) and had a minor fitness defect in the bladder (CI = 0.1, *P* = 0.031) (Fig. 4A).

During bacterial growth on gluconeogenic substrates, peptides and certain amino acids that are present in the urinary tract are broken-down into pyruvate, which can be oxidized in the TCA cycle or reduced to fermentative end-products. The resulting oxaloacetate can fuel gluconeogenesis as the substrate for pyruvate carboxykinase (*pckA*) that generates phophoenolpyruvate and bypasses the irreversible glycolytic reaction catalyzed by pyruvate kinase (*pykA*). Mutation of *pckA*, which disrupts gluconeogenesis, resulted in a significant fitness defect for *E. coli* in both bladder and kidneys (*P*<0.005) (Fig. 4A). Loss of *pckA* in *P. mirabilis* did not significantly affect colonization during urinary tract infection (Fig.4B). In support of differential utilization of amino acids present in the urinary tract, arginine and serine auxotrophs of *E. coli* demonstrate no fitness defect during UTI [8], while in *P. mirabilis*, serine auxotrophy created a 100-fold decrease in bladder colonization (CI = 10^−2^, *P*<0.005) (S2 Fig.).

The variable requirement for gluconeogenesis between both pathogens was not due to differences during *in vitro* culture; *pckA* mutant strains in both CFT073 and HI4320 backgrounds were indistinguishable from parental strains in LB medium and defined medium containing glucose (Fig. 4C and D). In *E. coli*, the *sdhB* mutant demonstrated a growth defect in LB medium (Fig. 4C) but not when cultured in defined glucose medium (Fig. 4E), while the *P. mirabilis frdA* mutant demonstrated a growth defect in defined glucose medium (Fig. 4F) but not when cultured in LB medium (Fig. 4D). Both *E. coli* and *P. mirabilis* with mutations in *fumC* demonstrated an *in vitro* growth defect in LB medium, but only the *P. mirabilis fumC* mutant was unable to replicate in defined glucose medium (Fig. 4F). Although both of these pathogenic isolates require components of the TCA cycle for fitness during infection, these *in vivo* and *in vitro* data suggest a key difference in respiration during growth on glycolytic substrates exists between *E. coli* and *P. mirabilis* despite both being enteric bacteria.

### Polymicrobial UTI shifts the fitness requirement for oxidative pentose phosphate from *P. mirabilis* to *E. coli*

**Figure 5:**
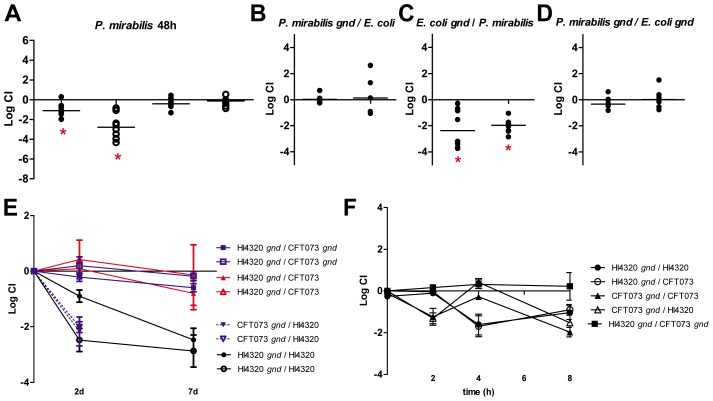
Polymicrobial infection alters central metabolism requirements for *E. coli* and *P. mirabilis*. (A) Competitive indices (CI) were determined following co-challenge infections of female CBA/J mice with a 1∶1 ratio of wild-type: mutant bacteria for *gnd* (oxidative pentose phosphate pathway) and *pckA* (gluconeogenesis) for *P. mirabilis* at 48 hpi. Each dot represents bladder (closed symbols) and kidneys (open symbols) from an individual animal. Competitive indices (CI) were determined 48 hpi for mixed infections of (B) wild-type *E. coli* CFT073 and *P. mirabilis* HI4320 *gnd*, (C) wild-type HI4320 and CFT073 *gnd*, and (D) HI4320 *gnd* and CFT073 *gnd* mutant constructs. Each circle represents bladder or kidneys from an individual animal. In (A–D) bars indicate the median CI and significant differences in colonization (*) (P<0.05) were determined by Wilcoxon signed-rank test. (E) *In vivo* CI at 48 h and 7 d post-infection. (F) CI during logarithmic growth in LB medium. For (A–F) a CI<1 indicates a fitness defect. For mixed infections CFU/ml were determined following plating of serial dilutions on LB agar with and without tetracycline. CFU from tetracycline-containing plates (*P. mirabilis* are Tet^R^) were subtracted from total CFU recovered on LB agar without antibiotics to determine CFU/ml for *E. coli* (Tet^S^).

The striking difference in the central pathway requirements during UTI between *E. coli* and *P. mirabilis* are puzzling because the central pathways are conserved and both pathogens are being assessed for fitness in the identical ascending UTI model. This suggests that an activity associated with the growth of the bacteria may cause alterations in the nutrient availability within the urinary tract. To test this, we performed mixed infections where the same mutation was tested against the opposite wild-type isolate. It was possible to distinguish *E. coli* from *P. mirabilis* by performing viable counts on agar with and without tetracycline due to *P. mirabilis* innate Tet^R^ phenotype. We chose to test the *gnd* mutants in both *E. coli* and *P. mirabilis* because that mutation created a severe fitness defect in *P. mirabilis* at 7 days in the bladder and kidneys, while no effect on fitness was observed for *E. coli* at 48 h in either tissue (Fig. 2A and B). In addition, the *P. mirabilis gnd* mutant demonstrated a fitness defect at 48 h in both the bladder and kidneys (Fig. 5A). Surprisingly, we found that when mixed 1∶1 with wild-type *E. coli* CFT073, the *P. mirabilis gnd* mutant was not out-competed at 48 h in either bladder or kidneys (Fig. 5B). Further, the *E. coli gnd* mutant was now significantly out-competed by the wild-type *P. mirabilis* HI4320 by>100-fold in both the bladder and kidneys (Fig. 5C). When the *gnd* mutants of each strain were mixed 1∶1, there was no observable difference in competitive indices (Fig. 5D). The same apparent reversal of *in vivo* fitness was also observed at 7 days post-inoculation (Fig. 5E), while no *in vitro* growth advantage was observed in any combination of *gnd* mutant bacteria and wild-type *E. coli* or *P. mirabilis* (Fig. 5F). Similarly, *E. coli* and *P. mirabilis* wild-type demonstrate equivalent growth during co-culture in LB medium and in defined medium with glucose as the sole carbon source (S3 Fig.).

### Polymicrobial infection with *E. coli* and *P. mirabilis* enhances bacterial colonization of the urinary tract

**Figure 6:**
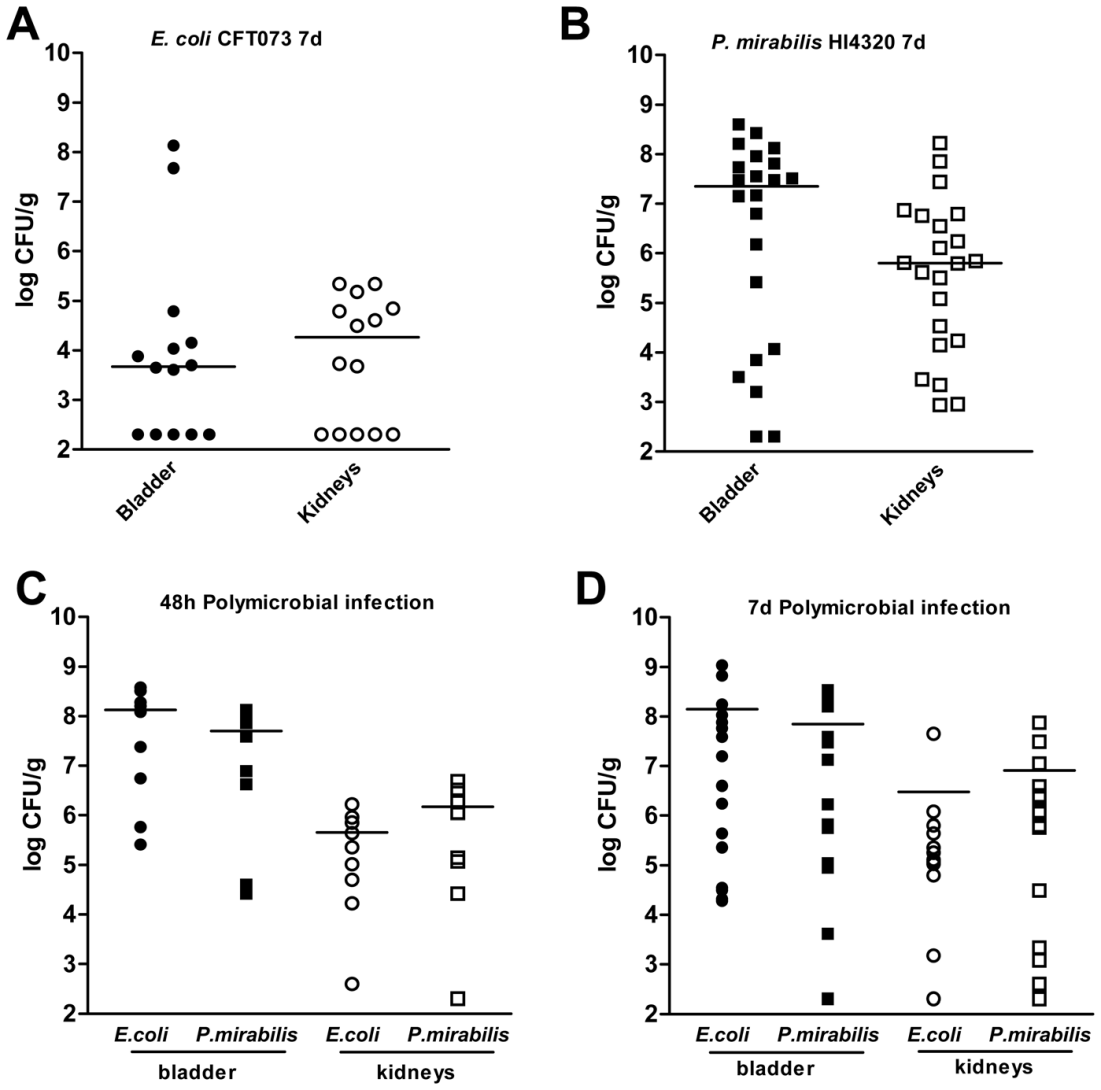
Polymicrobial urinary tract infection with *E. coli* and *P. mirabilis* enhances persistent bacterial colonization. Colonization levels following independent infections of female CBA/J mice with (A) UPEC strain CFT073 or (B) *P. mirabilis* HI4320. Colonization levels at (C) 48 hpi and (D) 7 dpi following polymicrobial infection of female CBA/J mice inoculated with a mixture of CFT073 and HI4320. The CFU/g of tissue for UPEC (circles) and *P. mirabilis* (squares) from bladders (closed symbols) and kidneys (open symbols) from individual animals at 48 hpi for (C) or 7 dpi for (A, B, D). Bars indicate the median values.

The observed differences in central pathways requirements between *E. coli* and *P. mirabilis* during colonization of the urinary tract led us to speculate that both species could co-exist within the same urinary tract without directly competing for nutrients. By performing mixed inoculations of *E. coli* and *P. mirabilis*, it was also possible to test whether the observed fitness reversal of the *gnd* mutant bacteria could be explained by intrinsic differences in the level of colonization during UTI. Indeed, as expected, the CFU/g of bladder and kidneys at 7 d are 2–3 logs higher for *P. mirabilis* than *E. coli* during single strain infection (Fig. 6A and B). When the wild-type *E. coli* CFT073 and *P. mirabilis* HI4320 were co-inoculated, however, the level of *E. coli* colonization in the bladder and kidneys increased by over 3 logs at both 48 h and 7 days post-inoculation (Fig. 6C and D) with a concomitant 10-fold increase in *P. mirabilis* colonization in both the bladder and kidneys (Fig. 6D). To further test the compatibility of these uropathogens we also performed sequential infections. Mice were inoculated with a single strain and colonization was established for 48 h prior to infecting with the second strain. In these sequential infections we observed that pre-colonization of the urinary tract did not exclude colonization by the second strain (S4 Fig.). Further, when *P. mirabilis* is used to infect and colonize mice, followed by infection with *E. coli* we observed enhanced colonization as seen when both bacteria are simultaneously co-inoculated into mice (S4 Fig.). These data demonstrate that mixed infection provides an obvious benefit for *E. coli* during UTI, but also that the presence of *E. coli* provides a mutual benefit by allowing *P. mirabilis* to colonize to a greater density than it would by itself.

## Discussion

The primary objective for microorganisms is to grow or replicate. For pathogenic microorganisms this need to replicate is paramount for their ability to successfully colonize, establish infection and cause disease. Many bacteria have evolved specific pathways that provide a growth advantage in a specific nutritional niche. These specific pathways often involve transport systems that aid the bacterium to acquire a certain nutrient, such as the ability of uropathogenic *E. coli* to import and utilize D-serine [31], [43] or the ability to sense α-ketoglutarate levels [44]. In contrast to these specific types of adaptations, most bacteria share highly conserved central pathways colloquially referred to as central metabolism. Our previous work began the comprehensive characterization of central pathways required by *E. coli* during UTI and found that gluconeogenesis and the TCA cycle were required to catabolize the dilute mixture of amino acids and peptides found in the urinary tract, while glycolysis, the pentose phosphate pathway, and the Entner Duodoroff pathway were dispensible [8]. In the present study, we extended this comprehensive study of *E. coli* central pathway requirements during UTI and in addition, performed parallel experiments using *P. mirabilis*, another well-studied uropathogen. We reasoned that both species of bacteria would have similar central metabolism requirements because they both occupy the same host niche, the urinary tract. Unexpectedly, we found that *E. coli* and *P. mirabilis* have strikingly divergent central pathway requirements despite infecting and growing in an identical host environment with presumably access to the same nutrients.

**Figure 7:**
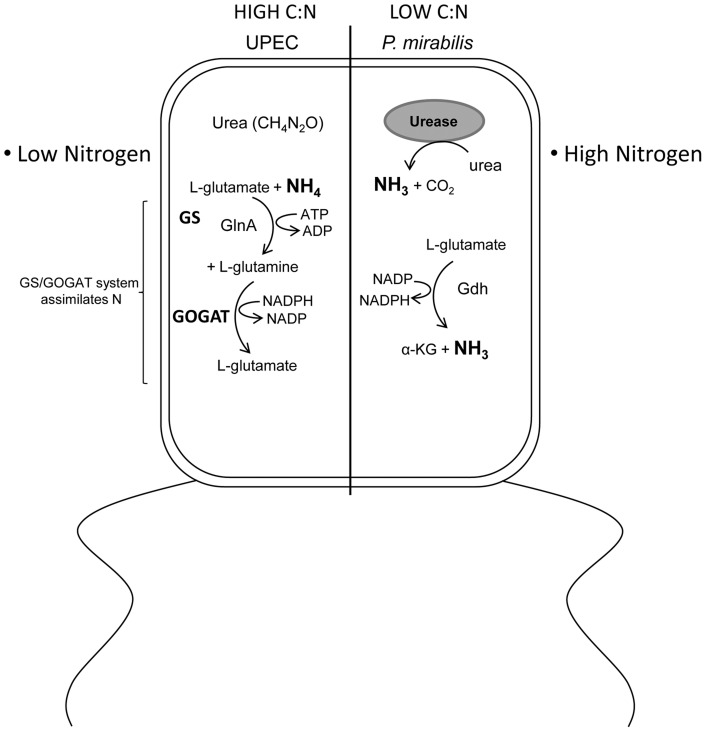
Model describing the differential effect of *E. coli* and *P. mirabilis* metabolism on the C/N ratio within the urinary tract. The urinary tract environment has a low C/N ratio due to the dilute mixture of amino acids and peptides as the primary carbon source and the abundance of urea in urine providing a substantial nitrogen contribution. *E. coli* is unable to utilize or sense the nitrogen sequestered in urea because it lacks urease, which liberates ammonia from urea. In contrast, *P. mirabilis* is urease positive; consequently, *P. mirabilis* senses a physiologically lower C/N ratio than *E. coli*. This results in *E. coli* activation of the glutamine synthetase and glutamate oxo-glutarate aminotransferase system (GS/GOGAT) to assimilate nitrogen while *P. mirabilis* assimilates nitrogen, via glutamate dehydrogenase (Gdh) due to the apparent excess nitrogen available from ammonia produced by urea hydrolysis. This difference in physiological nitrogen availability explains the dramatic difference between *E. coli* and *P. mirabilis* central carbon pathway requirements for fitness during urinary tract infection.

Our finding that *E. coli* and *P. mirabilis* have different central pathway requirements suggests that either a specific activity of the bacteria alters the host niche or that there is an intrinsic difference in the metabolic capabilities between the bacteria. Since we are studying highly conserved central pathways that are present in both species it is reasonable to conclude that a specific activity is present in one that is lacking in the other species. When considering the host urinary tract, the available nutrients are amino acids, peptides, and urea [40]. One obvious difference between *Proteus* and *E. coli* is that the former produces urease enzyme [45], [46], which hydrolyzes urea into ammonia and carbon dioxide (Fig. 7). The presence of urease activity would create a nitrogen rich environment by vastly increasing nitrogen availability via the concomitant increased ammonia concentration. In turn, the C/N ratio would be dramatically reduced for a urease producing bacteria like *P. mirabilis* relative to *E. coli* that does not have genes to encode urease. The altered C/N ratio would have profound effects on central pathway utilization because carbon metabolism and nitrogen assimilation is highly integrated [47], [48], [49]. Indeed, the apparent divergence in the ability to sense available nitrogen in urea results in *E. coli* activation of the glutamine synthetase and glutamate oxo-glutarate aminotransferase system (GS/GOGAT) to assimilate nitrogen [34], [35], while *P. mirabilis* assimilates nitrogen, via glutamate dehydrogenase (Gdh) [36] due to the apparent excess nitrogen available from ammonia produced by urea hydrolysis. We reason that this key difference is partly responsible for *P. mirabilis* requiring glycolysis, pentose phosphate pathway, and the ED pathway; while, on the other hand, the exact same mutations in *E. coli* have no affect on fitness during UTI. Alternatively, our findings raise the possibility that *E. coli* and *P. mirabilis* could reside in different cellular compartments. For example, it has been shown that *E. coli* can reside intracellularly during acute infection [50], [51], and while *P. mirabilis* can invade cultured cells [52], [53]; it is unclear if *P. mirabilis* spends a significant portion of the acute infection within host cells.

In addition to finding a remarkable difference in central metabolism requirements between two pathogens that infect the urinary tract, these findings have validated that glycolysis is dispensable for *E. coli* during UTI. In our previous work we assessed glycolysis by studying a mutation in the gene that encodes triose phosphate isomerase [8]; however, that enzyme is reversible. In the present study we created and tested phophofructokinase- and pyruvate kinase-deficient mutants in *E. coli* and *P. mirabilis*. These enzymes perform irreversible steps in glycolysis, thus by testing these mutations during UTI it is now clear that glycolysis is dispensible for *E. coli* and is required for *P. mirabilis* fitness *in vivo*. That glycolysis is required for *P. mirabilis* but not *E. coli* raises the possibility that sugars become available within the urinary tract during *Proteus* infection. That sugars become available when *P. mirabilis* colonizes the urinary tract would also allow for enhanced colonization of *E. coli* because sugars available during co-infection might increase growth of *E. coli* over numbers it would normally reach by solely consuming amino acids. This could also explain why *gnd* mutant *E. coli* displayed a fitness defect only when co-colonized with *P. mirabilis*.

It has also been shown that the oxidative TCA cycle is important for *E. coli* fitness during UTI [8], [42], but whether or not the reductive TCA cycle is important for fitness during UTI remains unanswered. We found that mutants lacking fumarate reductase, which is an enzyme that utilizes fumarate as an electron acceptor during anaerobic respiration, do not have a fitness defect in *E. coli* as predicted by our earlier study. In contrast, fumarate reductase mutants in *P. mirabilis* are out-competed by wild-type. These data show that the branched, reductive TCA pathway is not important for *E. coli* to grow within the urinary tract, supporting the notion that the urinary tract is moderately oxygenated [35]. However, the result that fumarate reductase is important for *P. mirabilis* fitness during UTI suggests an anaerobic environment does exist in the host urinary tract. In addition, because sensing oxygen depletion has been shown to be important for *E. coli* fitness during UTI [44], it remains possible that fumarate reductase is dispensible for *E. coli* due to the presence of alternative energy pathways like Ni-Fe hydrogenases and nitrate reductase, these and other redox enzymes are part of the large repertoire of electron transfer components available for the highly modular respiratory chains that can be assembled in *E. coli* [54]. Future studies will be useful to determine the exact energy pathways used by *E. coli* during UTI.

The urogenital track of humans is considered a site of polymicrobial colonization [55] and it has been shown that UTI can be polymicrobial [56], including co-infections with *E. coli* and *P. mirabilis* [57], [58]. Polymicrobial UTI has also been suggested to enhance *E. coli* virulence [58]. Our findings that two preeminent UTI pathogens have divergent central metabolism requirements have important implications for polymicrobial infections. Specifically, these data indicate that *E. coli* and *P. mirabilis* may not directly compete for nutrients during colonization of the urinary tract and led us to hypothesize that co-inoculation of *E. coli* and *P. mirabilis* may lead to enhanced colonization levels during experimental UTI. Indeed, we found that colonization was enhanced for both *E. coli* and *P. mirabilis*. The benefit of polymicrobial infection was more apparent for *E. coli* over time. In addition to supporting that *E. coli* and *P. mirabilis* may not directly compete for resources during UTI, these findings could alternatively suggest that some activity of one or both pathogenic bacteria may alter the host niche in such a way that facilitates bacterial replication. In support of this, we observed that *P. mirabilis* causes more tissue damage and larger induction of the pro-inflammatory cytokines IL-1α, IL-β, IL-6, and G-CSF than does *E. coli* in independent infection, while a mixed infection appears more similar to *E. coli* alone (S5 Fig.). With a systematic view of bacterial metabolism during UTI in hand, further studies of the host innate response to these infections will help form a comprehensive view of how the host response shapes bacterial carbon utilization during UTI.

To test whether or not the bacteria differences in the host urinary tract niche, we decided to co-inoculate *E. coli* and *P. mirabilis* mutants with the heterologous wild-type strain and assess fitness during UTI. Our findings showed that polymicrobial infection of the urinary tract changed the fitness results when compared to mono-species infection. The *E. coli gnd* mutant, which was not outcompeted by its parent strain, demonstrated a colonization disadvantage when co-inoculated with *P. mirabilis* wild-type. Conversely, the *P. mirabilis gnd* mutant, which was out-competed by its parent strain, did not demonstrate a disadvantage in colonization when co-inoculated with wild-type *E. coli*. Although future studies with additional mutants will be useful to further elucidate how co-inoculation might change central pathway requirements, these data do suggest that the host niche environment is altered by bacterial activity during UTI. Importantly, our study leads to a more complete picture of the metabolism of two key bacterial pathogens that cause UTI and shows that polymicrobial infection of the urinary tract may alter the metabolic pathways required for optimal growth within the host. These findings provide a better understanding of bacterial metabolism during clinically relevant infections and represent an important foundation to begin to dissect how metabolism and virulence intersect during UTI and how polymicrobial interactions may affect pathogenesis of extraintestinal *E. coli* infections.

## Materials and Methods

### Bacterial strains and culture conditions

*P. mirabilis* HI4320 was isolated from urine of a patient presenting with bacteriuria during long-term catheterization [56], [59]. *E. coli* CFT073 was isolated from the blood and urine of a patient with acute pyelonephritis [37]. *E. coli* and *P. mirabilis* were routinely cultured in lysogeny broth (LB) medium. For growth experiments, wild-type and mutant strains of *E. coli* and *P. mirabilis* were cultured in MOPS defined medium [60] and minimal salts medium [61], respectively, both containing either 0.2% (w/v) glucose or 0.2% (w/v) glycerol as the sole carbon source. Defined medium cultures were inoculated 1∶50 and LB cultures were inoculated 1∶100 from overnight LB bacterial cultures and incubated with aeration at 37°C. Growth curves were performed in triplicate; OD_600_ was recorded every hour.

### Construction of metabolism mutants

*P. mirabilis* HI4320 mutants (S1 Table) were generated using the TargeTron Gene Knockout System (Sigma). Oligonucleotides for mutant construction were created using the TargeTron Design Site (Sigma). PCR confirmation of mutants was performed using oligonucleotides flanking the intron insertion site designed with the PrimerQuest program on the Integrated DNA Technologies website. *E. coli* CFT073 deletion mutants (S1 Table) were constructed using the lambda red recombinase system [62]. Primers homologous to sequences within the 5′ and 3′ ends of the target genes were designed and used to replace target genes with a nonpolar kanamycin- or chloramphenicol-resistance cassette derived from the template plasmid pKD4 or pKD3, respectively [62]. Confirmation of *E. coli* mutants was carried out by PCR using primers flanking the target gene sequence and comparing product size to wild-type PCR product size. When size differences were negligible PCR products were digested with a restriction enzyme (New England Biolabs).

### Complementation of mutants

For *in vitro* complementation, the *tpiA* gene was amplified from *P. mirabilis* genomic DNA using Easy-A high fidelity polymerase (Stratagene) and independently cloned into pGEN-MCS [63], [64] using appropriate restriction enzymes. The sequences of pGEN-*tpiA* were verified by DNA sequence analysis prior to electroporation into the *P. mirabilis tpiA* mutant strain and *E. coli tpiA* mutant strain.

### Experimental UTI

The CBA mouse model of ascending UTI [39], [65] was used to assess the fitness contribution of each metabolic mutant during co-challenge competition. To determine persistence of wild-type strains, independent infections of a single strain were performed. Female CBA/J mice (6–8 week old; 20 to 22 g; Jackson Laboratories) were anesthetized with ketamine/xylazine and transurethrally inoculated with a 50 µl bacterial suspension (total inoculum  = 5×10^7^ or 2×10^8^ CFU) per mouse using a sterile polyethylene catheter (I.D. 0.28 mm × O.D. 0.61 mm) connected to an infusion pump (Harvard Apparatus). For *in vivo* co-challenges, a suspension containing 5×10^7^ CFU of a 1∶1 ratio of *P. mirabilis* HI4320 and *P. mirabilis* kanamycin-resistant mutant in LB medium or a suspension containing 2×10^8^ CFU of a 1∶1 ratio of *E. coli* CFT073 and *E. coli* antibiotic-resistant mutant in PBS. For independent infections, the respective *P. mirabilis* or *E. coli* suspensions contained only the wild-type strain. Input CFU/ml was determined by plating serial dilutions (Spiral Biotech) of each inoculum onto low salt (0.5 g NaCl/L) LB agar, to prevent *P. mirabilis* swarming, with and without antibiotic. For experiments with *P. mirabilis*, low salt LB agar (0.5 g NaCl/L) was used to prevent swarming. Infected mice were euthanized 48 h or 7 d post infection, bladder and kidneys were aseptically removed, weighed, and homogenized (OMNI International) in 3 ml PBS, and appropriate dilutions were spiral plated on LB agar with and without antibiotic to determine the output CFU/g of tissue. Viable counts were enumerated using QCount software (Spiral Biotech) and CFU from antibiotic-containing medium (mutant CFU) were subtracted from the total CFU from plates lacking antibiotic to determine the number of wild-type bacteria. For co-challenge experiments, competitive indices (CI) were calculated by dividing the ratio of the CFU of mutant to wild-type recovered from each mouse following infection by the ratio of the CFU of mutant to the CFU of wild-type present in the input. CI data were log-transformed and analyzed by the Wilcoxon signed-rank test to determine statistically significant differences in colonization (*P*-value <0.05). A CI>1 indicates that the mutant out-competes the wild-type strain and a CI<1 indicates that the mutant is out-competed by the wild-type strain. For independent infections the Mann-Whitney test was used to determine statistically significant differences in colonization (*P*-value <0.05).

### Polymicrobial infections

The relative fitness *in vivo* for bacteria during polymicrobial infection was determined by co-inoculating UPEC CFT073 and *P. mirabilis* HI4320 strains and deletion mutants into the same female CBA mice as described previously with the following modification. For polymicrobial co-challenge infections, a 1∶1 (v/v) mixture was prepared containing 2.5×10^7^ CFU of *P. mirabilis* HI4320 in LB medium and 10^8^ CFU *E. coli* CFT073 in PBS. Competitive indices were calculated as described above. For polymicrobial infections containing only wild-type strains, the CFU/g tissue were determined following plating of serial dilutions on low salt (0.5 g NaCl/L) LB agar with and without tetracycline; *P. mirabilis* is intrinsically tetracycline-resistant. CFU from tetracycline agar plates, which represent *P. mirabilis*, were subtracted from total CFU recovered on LB agar without antibiotics to determine CFU/g for *E. coli*. For quantification of bacteria recovered from a polymicrobial infection with a wild-type strain and a heterologous kanamycin-resistant mutant strain, CFU on LB agar containing kanamycin (mutant) were subtracted from total CFU recovered on LB without antibiotics, to determine wild-type CFU/g tissue. Tetracycline was used to enumerate bacterial colonization levels following polymicrobial infection with heterologous kanamycin-resistant strains.

### Ethics statement

All animal experiments were performed in accordance to the protocol (08999-3) approved by the University Committee on Use and Care of Animals at the University of Michigan. This protocol is in complete compliance with the guidelines for humane use and care of laboratory animals mandated by the National Institutes of Health.

## Supporting Information

S1 Fig ***In vitro*** **growth of pentose phosphate and Entner-Doudoroff pathway mutants.** Growth of (A, C) UPEC CFT073 and (B, D) *P. mirabilis* HI4320 wild-type strains and mutants in: *gnd*, 6-phosphogluconate dehydrogenase; *talB*, transaldolase; and *edd*, 6-phosphogluconate dehydratase in LB medium (A, B) or defined medium containing 0.2% glucose (C, D) as the carbon source. A representative growth curve is shown for each panel.
(TIF)

S2 Fig ***In vivo*** **contribution of arginine and serine biosynthesis.** (A, B) Competitive indices (CI) were determined following co-challenge infections with female CBA/J mice with a 1∶1 ratio of either wild-type (A) *E. coli* CFT073 or (B) *P. mirabilis* HI4320 and their respective mutants in: *argG*, argininosuccinate dehydrogenase and *serA*, D-3-phosphglycerate dehydrogenase. UPEC was recovered at 48 h post-inoculation. *P. mirabilis* was recovered at 7 d post-inoculation. Each dot represents bladder (closed symbols) and kidneys (open symbols) from an individual animal. Bars indicate the median CI. Significant differences in colonization (*P<0.05) were determined by the Wilcoxon signed-rank test. A CI<1 indicates a fitness defect. Growth of *P. mirabilis* HI4320 wild-type strain and amino acid auxotroph mutants in (C) LB medium and (D) defined medium containing 0.2% glucose with or without 10 mM of the indicated amino acid.
(TIF)

S3 Fig ***In vitro*** **co-culture of wild-type** ***E. coli*** **CFT073 and** ***P. mirabilis*** **HI4320.** A 1∶1 ratio containing 10^6^ CFU/ml of each strain was used to inoculate LB medium (solid symbols) and minimal salts medium containing 0.2% glucose (open symbols). Co-cultures were incubated at 37°C with agitation for 24 h; log CFU/ml were determined following plating of serial dilutions on LB agar with and without tetracycline. CFU from tetracycline-containing plates (*P. mirabilis*) were subtracted from total CFU recovered on LB agar without antibiotics to determine CFU/ml for *E. coli* (Tet^S^).
(TIF)

S4 Fig ***E. coli*** **colonization of the urinary tract is enhanced following pre-colonization by** ***P. mirabilis*** **during sequential infection.** Colonization levels of (A) *E. coli* CFT073 or (B) *P. mirabilis* HI4320 following sequential *in vivo* co-infections. On day zero (_0_) female CBA/J mice were infected with either wild-type strain *E. coli* CFT073 or *P. mirabilis* HI4320. Following 2 days (_2_) mice were inoculated with the other wild-type species. Mice were euthanized and organs collected 48 h following the secondary infection. Each dot represents log_10_ CFU/g tissue of bladder or kidneys from an individual animal. The bars indicate median log_10_ CFU for bladder and kidneys. CFU/ml were determined following plating of serial dilutions on LB agar with and without tetracycline (15 µg/ml). CFU from Tet plates (*P. mirabilis*) was subtracted from total CFU recovered on LB agar without antibiotics to determine CFU/ml for *E. coli* (Tet^S^). *P*-values indicated on the graph were determined by the Mann-Whitney test.
(TIF)

S5 Fig **Urinary tract pathology and urinary proinflammatory cytokine production during mono-species and polymicrobial infection.** Groups of 5 mice (*n* = 15) were inoculated with *E. coli* CFT073 alone, *P. mirabilis* HI4320 alone, or a 1∶1 mixture of both *E. coli* and *P. mirabilis*. (A) Inflammation and lesions were scored for pyelonephritis, peripelvic inflammation, and cystitis Tissue pathology was scored from 0–3, with 3 being most severe. Severity was determined by assessing for the presence of PMNs and lymphocytes, focal aggregates of PMNs or lymphocytes, edema, and transmural inflammation. (B) Urine was collected from all 15 mice before inoculation (uninfected control) and from infected mice at 24, 48, 72, and 96 h post-inoculation. Urine from each group were pooled and used to analyze a panel of 12 pro-inflammatory cytokines by ELISA.
(TIF)

S1 Table **Bacterial strains used in this study.**
(DOCX)
