# Supplementary figures and images for: Preferential Use of Central Metabolism In Vivo Reveals a Nutritional Basis for Polymicrobial Infection
Source: PLoS Pathog. 2015 Jan 8;11(1):e1004601. doi: 10.1371/journal.ppat.1004601 (PMC4287612; doi:10.1371/journal.ppat.1004601)

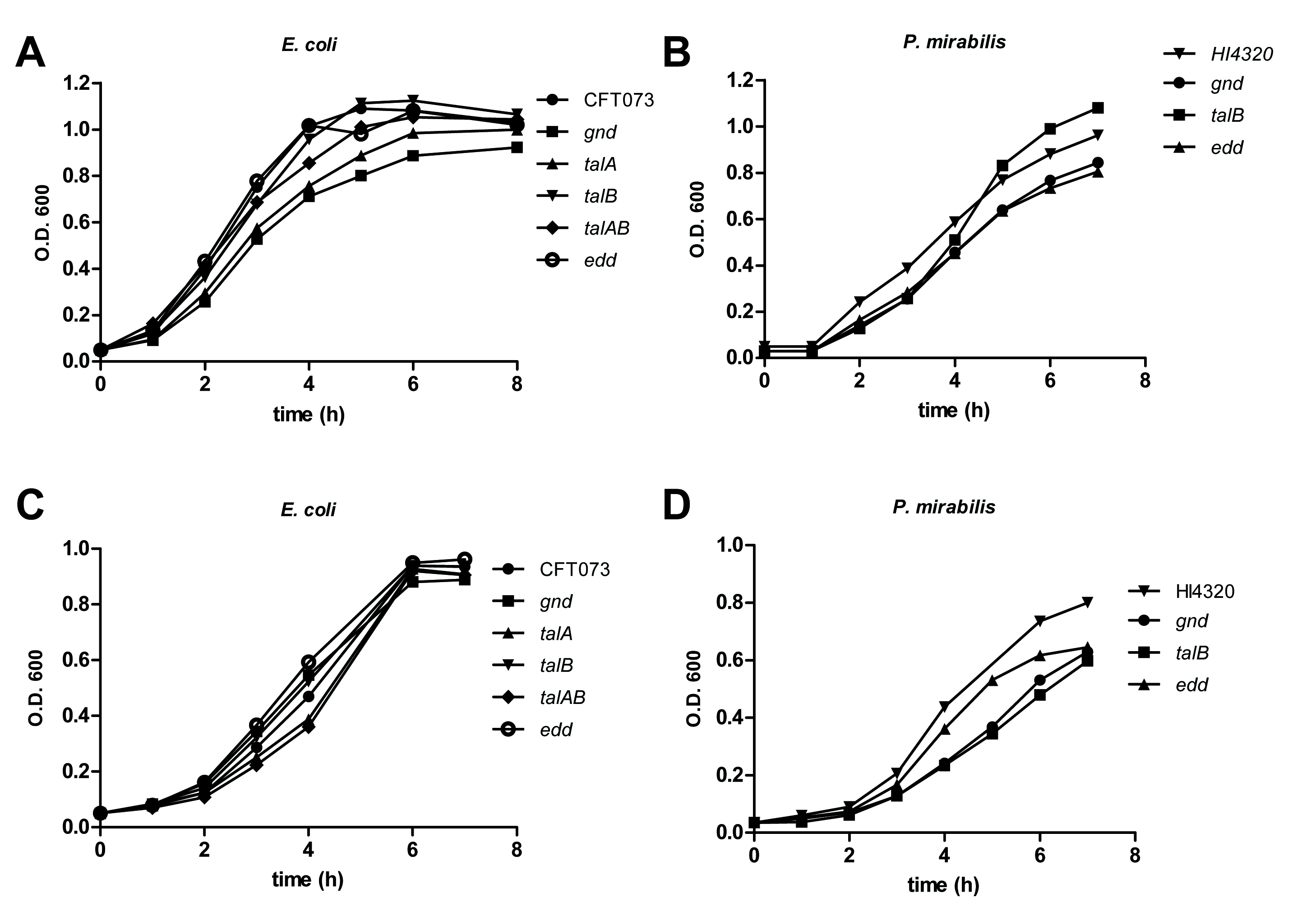

Supplement: S1 Fig — In vitro growth of pentose phosphate and Entner-Doudoroff pathway mutants. Growth of (A, C) UPEC CFT073 and (B, D) P. mirabilis HI4320 wild-type strains and mutants in: gnd, 6-phosphogluconate dehydrogenase; talB, transaldolase; and edd, 6-phosphogluconate dehydratase in LB medium (A, B) or defined medium containing 0.2% glucose (C, D) as the carbon source. A representative growth curve is shown for each panel. (TIF) [file ppat.1004601.s001.tif]

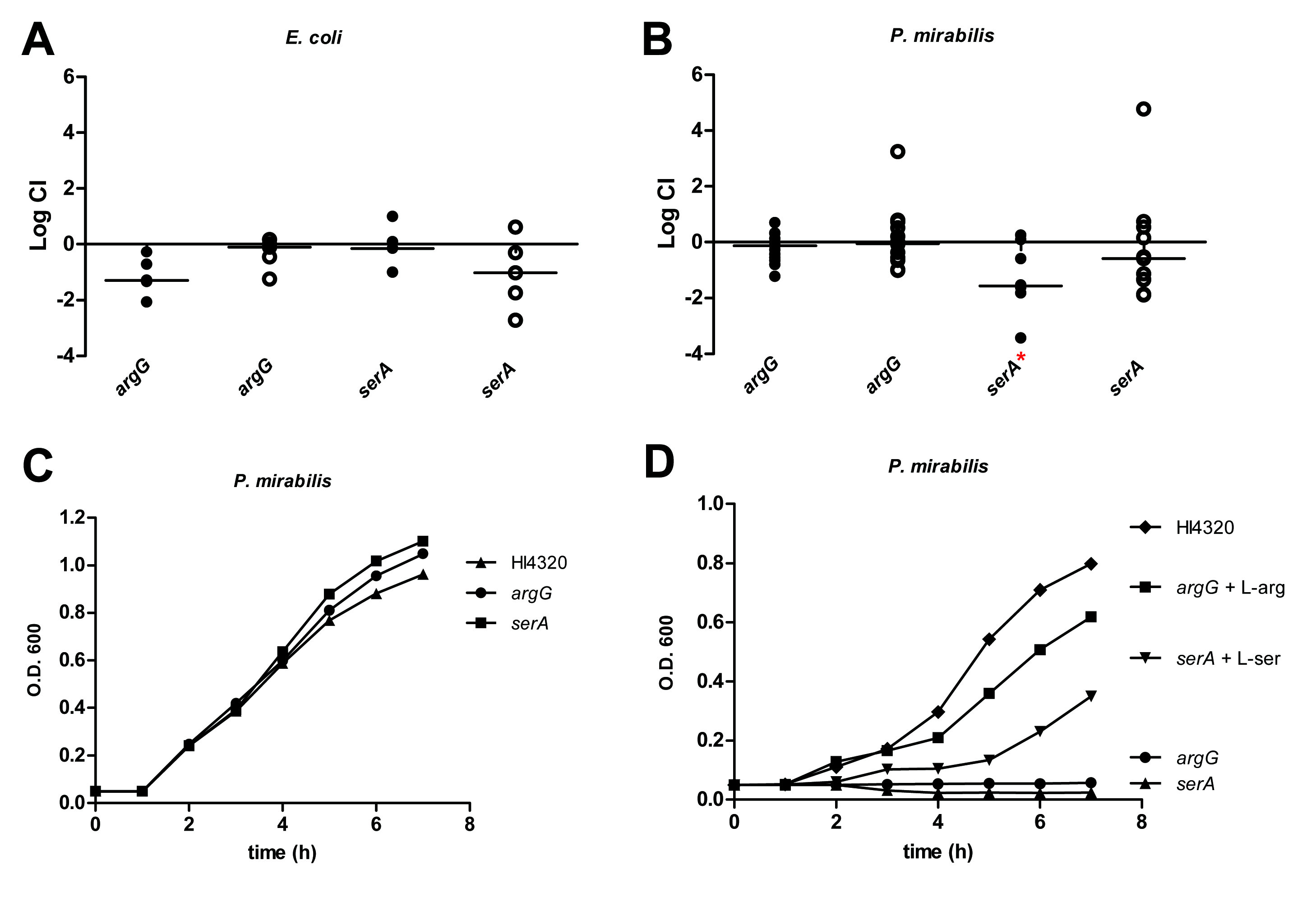

Supplement: S2 Fig — In vivo contribution of arginine and serine biosynthesis. (A, B) Competitive indices (CI) were determined following co-challenge infections with female CBA/J mice with a 1∶1 ratio of either wild-type (A) E. coli CFT073 or (B) P. mirabilis HI4320 and their respective mutants in: argG, argininosuccinate dehydrogenase and serA, D-3-phosphglycerate dehydrogenase. UPEC was recovered at 48 h post-inoculation. P. mirabilis was recovered at 7 d post-inoculation. Each dot represents bladder (closed symbols) and kidneys (open symbols) from an individual animal. Bars indicate the median CI. Significant differences in colonization (*P<0.05) were determined by the Wilcoxon signed-rank test. A CI<1 indicates a fitness defect. Growth of P. mirabilis HI4320 wild-type strain and amino acid auxotroph mutants in (C) LB medium and (D) defined medium containing 0.2% glucose with or without 10 mM of the indicated amino acid. (TIF) [file ppat.1004601.s002.tif]

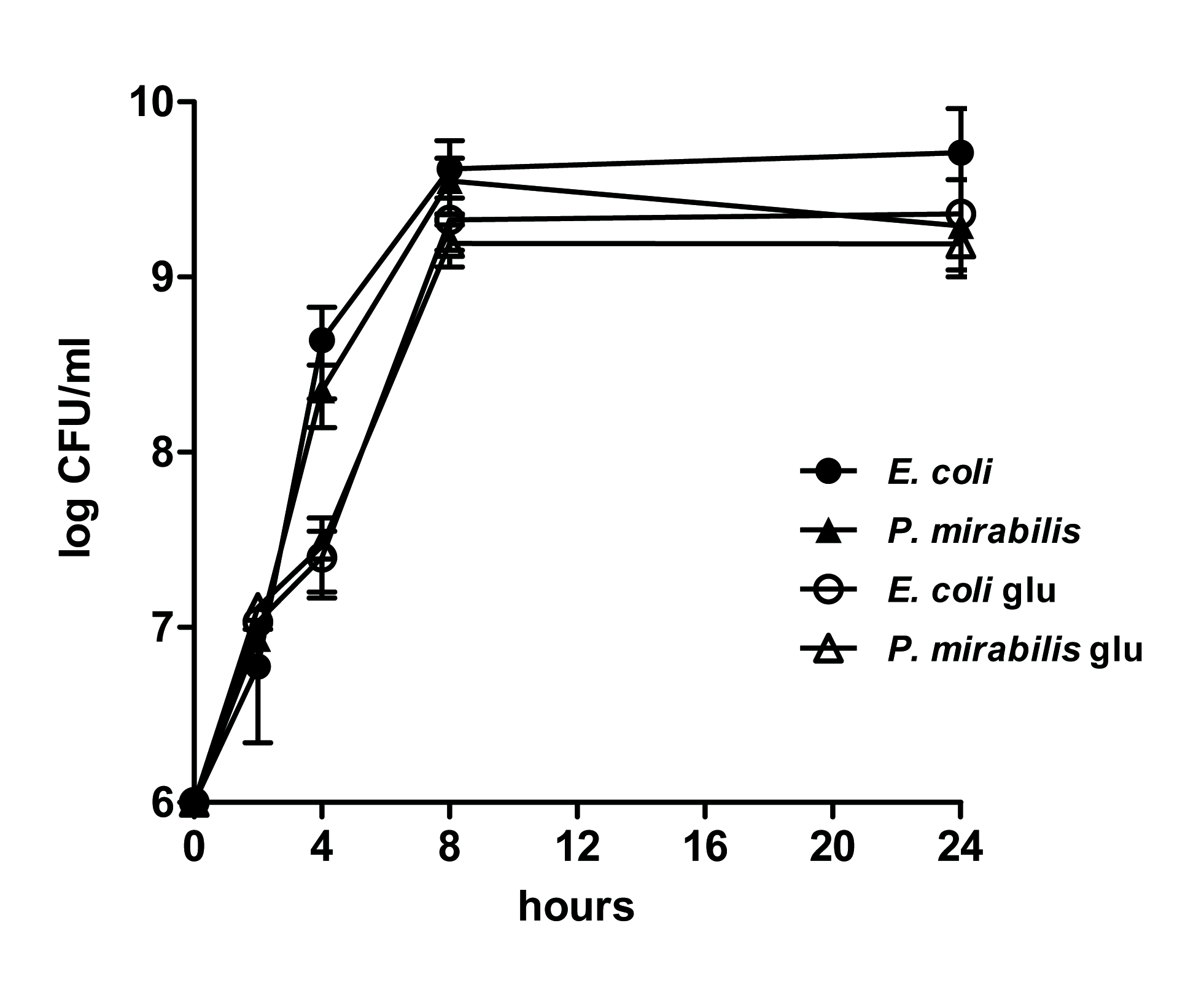

Supplement: S3 Fig — In vitro co-culture of wild-type E. coli CFT073 and P. mirabilis HI4320. A 1∶1 ratio containing 106 CFU/ml of each strain was used to inoculate LB medium (solid symbols) and minimal salts medium containing 0.2% glucose (open symbols). Co-cultures were incubated at 37°C with agitation for 24 h; log CFU/ml were determined following plating of serial dilutions on LB agar with and without tetracycline. CFU from tetracycline-containing plates (P. mirabilis) were subtracted from total CFU recovered on LB agar without antibiotics to determine CFU/ml for E. coli (TetS). (TIF) [file ppat.1004601.s003.tif]

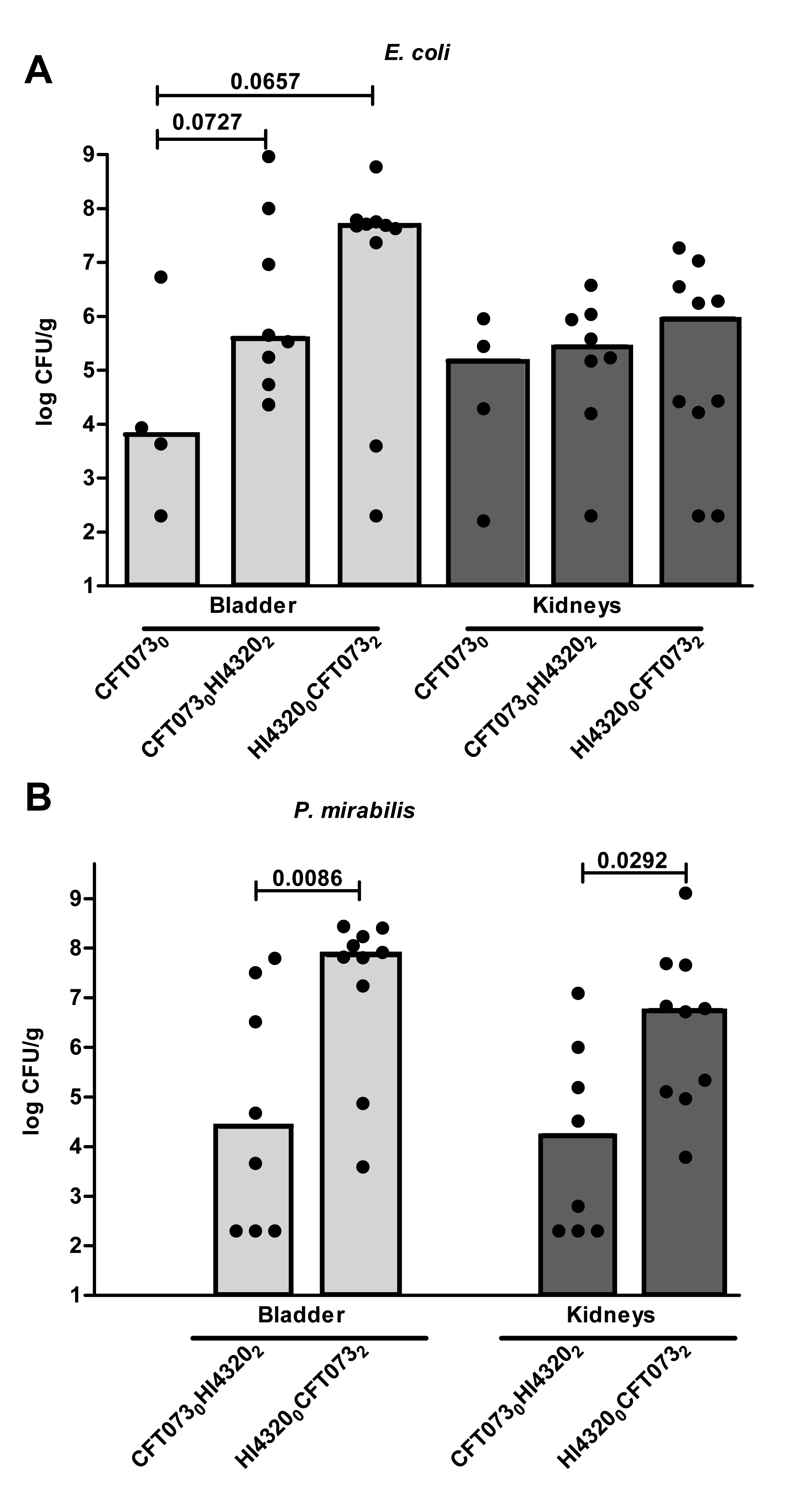

Supplement: S4 Fig — E. coli colonization of the urinary tract is enhanced following pre-colonization by P. mirabilis during sequential infection. Colonization levels of (A) E. coli CFT073 or (B) P. mirabilis HI4320 following sequential in vivo co-infections. On day zero (0) female CBA/J mice were infected with either wild-type strain E. coli CFT073 or P. mirabilis HI4320. Following 2 days (2) mice were inoculated with the other wild-type species. Mice were euthanized and organs collected 48 h following the secondary infection. Each dot represents log10 CFU/g tissue of bladder or kidneys from an individual animal. The bars indicate median log10 CFU for bladder and kidneys. CFU/ml were determined following plating of serial dilutions on LB agar with and without tetracycline (15 µg/ml). CFU from Tet plates (P. mirabilis) was subtracted from total CFU recovered on LB agar without antibiotics to determine CFU/ml for E. coli (TetS). P-values indicated on the graph were determined by the Mann-Whitney test. (TIF) [file ppat.1004601.s004.tif]
