# Supplementary material for: Preferential Use of Central Metabolism In Vivo Reveals a Nutritional Basis for Polymicrobial Infection
Source: PLoS Pathog. 2015 Jan 8;11(1):e1004601. doi: 10.1371/journal.ppat.1004601 (PMC4287612; doi:10.1371/journal.ppat.1004601)
Supplement: S1 Table — Bacterial strains used in this study. (DOCX) [file ppat.1004601.s006.docx]

| Table S1. Bacterial strains used in this study. | |  |
| --- | --- | --- |
| Strain | Genotype | Source |
| *E. coli* strains |  |  |
| CFT073 | Wild-type pyelonephritis isolate (O6:K2:H1) | [37] |
| *pfkA* | CFT073 ∆*pfkA* (Kan^r^) | This study |
| *pgi* | CFT073 ∆*pgi* (Kan^r^) | [8] |
| *tpiA* | CFT073 ∆*tpiA* (Kan^r^) | [8] |
| *pykA* | CFT073 ∆*pykA* (Kan^r^) | This study |
| *gnd* | CFT073 ∆*gnd* (Kan^r^) | [8] |
| *talB* | CFT073 ∆*talB* (Cam^r^) | This study |
| *talA* | CFT073 ∆*talA* (Kan^r^) | [8] |
| *talAtalB* | CFT073 ∆*talAtalB* (Kan^r^Cam^r^) | This study |
| *edd* | CFT073 ∆*edd* (Kan^r^) | [8] |
| *sdhB* | CFT073 ∆*sdhB* (Kan^r^) | [8] |
| *frdA* | CFT073 ∆*frdA* (Cam^r^) | This study |
| *fumC* | CFT073 ∆*fumC* (Kan^r^) | This study |
| *pckA* | CFT073 ∆*pckA* (Kan^r^) | [8] |
| *P. mirabilis* strains |  |  |
| HI4320 | Wild-type bacteriuria isolate (Tet^r^) | [59] |
| *pfkA* | HI4320 *pfkA::kan* | This study |
| *pgi* | HI4320 *pgi::kan* | This study |
| *tpiA* | HI4320 *tpiA::kan* | This study |
| *pykA* | HI4320 *pykA::kan* | This study |
| *gnd* | HI4320 *gnd::kan* | This study |
| *talB* | HI4320 *talB::kan* | This study |
| *edd* | HI4320 *edd::kan* | This study |
| *sdhB* | HI4320 *sdhB::kan* | This study |
| *frdA* | HI4320 *frdA::kan* | This study |
| *fumC* | HI4320 *fumC::kan* | This study |
| *pckA* | HI4320 *pckA::kan* | This study |
| *argG* | HI4320 *argG::kan* | This study |
| *serA* | HI4320 *serA::kan* | This study |
| Plasmids |  |  |
| pGEN-MCS | Low copy number plasmid (Amp^r^) | [8] |
| pGEN-*tpiA* | pGEN-MCS digested with EcoRI and HindIII replacing MCS site with *tpiA* (Amp^r^) | This study |
